# Lipopolysaccharide induces mitochondrial dysfunction in rat cardiac microvascular endothelial cells

**DOI:** 10.1186/cc9665

**Published:** 2011-03-11

**Authors:** M Vuda, M Chiusa, SM Jakob, J Takala, C Zuppinger, S Djafarzadeh

**Affiliations:** 1 Bern University Hospital and University of Bern, Switzerland

## Introduction

Endothelial injury and dysfunction are key patho-physiological processes in sepsis. The aim of the study was to evaluatethe effects of bacterial lipopolysaccharide (LPS) on cellular respiration of rat primary cardiac microvascular endothelial cells (CMEC).

## Methods

CMEC were isolated from adult (250 to 300 g) male Wistar rats and cultured. Cells were exposed to LPS (1 μg/ml) for 4, 8, 16 hours and cellular respiration was measured by high-resolution respirometry (Oxygraph-2k; Oroboros Instruments, Innsbruck, Austria). Activation of caspase-3 protein as an early apoptotic event was examined by western blot analysis. Electron microscopy was performed to reveal any alterations in mitochondrial morphology.

## Results

**Figure 1:**
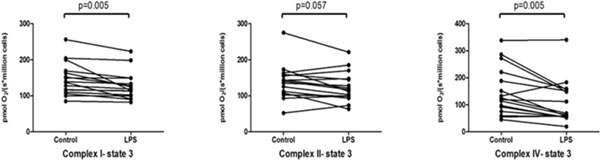
**Cardiac microvascular endothelial cells' oxygen consumption**.

**Figure 2:**
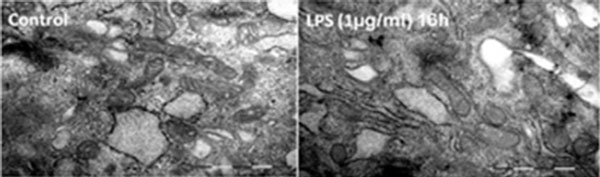
**CMEC cells under control and LPS treatment conditions**.

After 4 and 8 hours of LPS incubation (1 μg/ml) no significant changes in CMEC mitochondrial respiration was observed. However, cells treated with LPS for 16 hours exhibited a significant reduction in the maximal complex I-dependent (control: 146 ± 45 pmol/(second*million cells) vs. LPS: 127 ± 38 pmol/(second*million cells)) and IV-dependent (control:148 ± 89 pmol/(second*million cells) vs. LPS: 108 ± 80 pmol/(second*million cells)) mitochondrial respiration (*n *= 16) (Figure 1). Relatively little, if any, processing of procaspase-3 to active caspase-3 was detected in untreated cells or in cells treated with LPS (1 μg/ml, 16 hours of incubation) (data not shown), and electron microscopy examination revealed no major alterations in cellular and mitochondrial ultrastucture under LPS treatment (Figure 2). Statistical analysis for cellular respiration was performed using a paired *t *test.

## Conclusions

The data suggest that prolonged exposure to LPS impairs CMEC complex I-dependent and IV-dependent respiration slightly but significantly, without apparent signs of apoptosis or mitochondrial ultrastructural damage.

